# Application of antimicrobial peptides in plant protection: making use of the overlooked merits

**DOI:** 10.3389/fpls.2023.1139539

**Published:** 2023-07-19

**Authors:** Rui Tang, Hui Tan, Yan Dai, Lin’ai Li, Yan Huang, Huipeng Yao, Yi Cai, Guozhi Yu

**Affiliations:** College of Life Sciences, Sichuan Agricultural University, Yaan, China

**Keywords:** plant protection, antimicrobial peptide (AMP), phytopathogen, rapid killing, resistance evolution, synergism

## Abstract

Pathogen infection is one of the major causes of yield loss in the crop field. The rapid increase of antimicrobial resistance in plant pathogens has urged researchers to develop both new pesticides and management strategies for plant protection. The antimicrobial peptides (AMPs) showed potential on eliminating plant pathogenic fungi and bacteria. Here, we first summarize several overlooked advantages and merits of AMPs, which includes the steep dose-response relations, fast killing ability, broad synergism, slow resistance selection. We then discuss the possible application of AMPs for plant protection with above merits, and highlight how AMPs can be incorporated into a more efficient integrated management system that both increases the crop yield and reduce resistance evolution of pathogens.

## Introduction

Since 1950s, antibiotics have been used to control bacterial and fungal pathogens in the crop field. The crisis of antibiotic resistance in agricultural system is looming. Overuse of antibiotics in crop production has dramatically accelerated the evolution of antibiotic resistance of phytopathogen (McManus et al., 2002). The bacterial genera of plant pathogen, which includes *Erwinia, Pantoea, Pseudomonas, Xanthomonas*, acquired high resistance to streptomycin and oxytetracycline during the past decades (Sundin and Wang, 2018). The increasing resistance of pathogens indirectly plied up overall cost for plant protection. It also raised great concern that resistant bacterial mutants will fail the clinical treatment. For example, several phytopathogenic fungi have evolved high resistance to azole, a class of anti-fungal drug used both in farmland and hospital (Dunne et al., 2017).

In order to suppress the increasing trend of antibiotic resistance in plant protection, researchers have focused on either discovering new antimicrobials or developing new strategies for fighting against plant pathogens (Marcos et al., 2008; Lindsey et al., 2020). In recent years, a large number of natural and synthetic new chemicals are discovered and used in plant protection. Among them, AMPs are good candidates for fighting against phytopathogen (Amso and Hayouka, 2019). Moreover, new ways of drug application with existing antibiotics were also developed based on precisely quantitative methods, which significantly enhanced treatment efficiency and delayed the resistance evolution. For example, streptomycin is re-registered for treating Huanglongbing in the citrus grove. With methods analogues to the pharmacokinetics in pharmacology, recent studies have determined its spatial and temporal variation of drug concentration inside the plant. This helps to identify effective concentration range and duration of effectiveness inside citrus trees (Li et al., 2021). In addition, the patterns of killing of antibiotics are also critical for evaluation of field efficacy of antibiotics. It has been suggested that benchmark dose modeling can be applied to determine the killing effect of pesticides both in laboratory and field in the framework of the dose-response relation (Jensen et al., 2019, Jensen et al., 2022). In fact, this method is akin to pharmacodynamics in pharmacology. Pharmacodynamics of antibiotic provide information on how fast a given drug kills bacteria with respect to the dose. Moreover, dose-response relation also characterizes the ability of resistance selection of the antimicrobials (Yu et al., 2018). Overall, integration of new drug and novel methods of application facilitate the development of integrated pest management, reducing resistance evolution and cutting the total cost of agricultural production.

**Figure 1:**
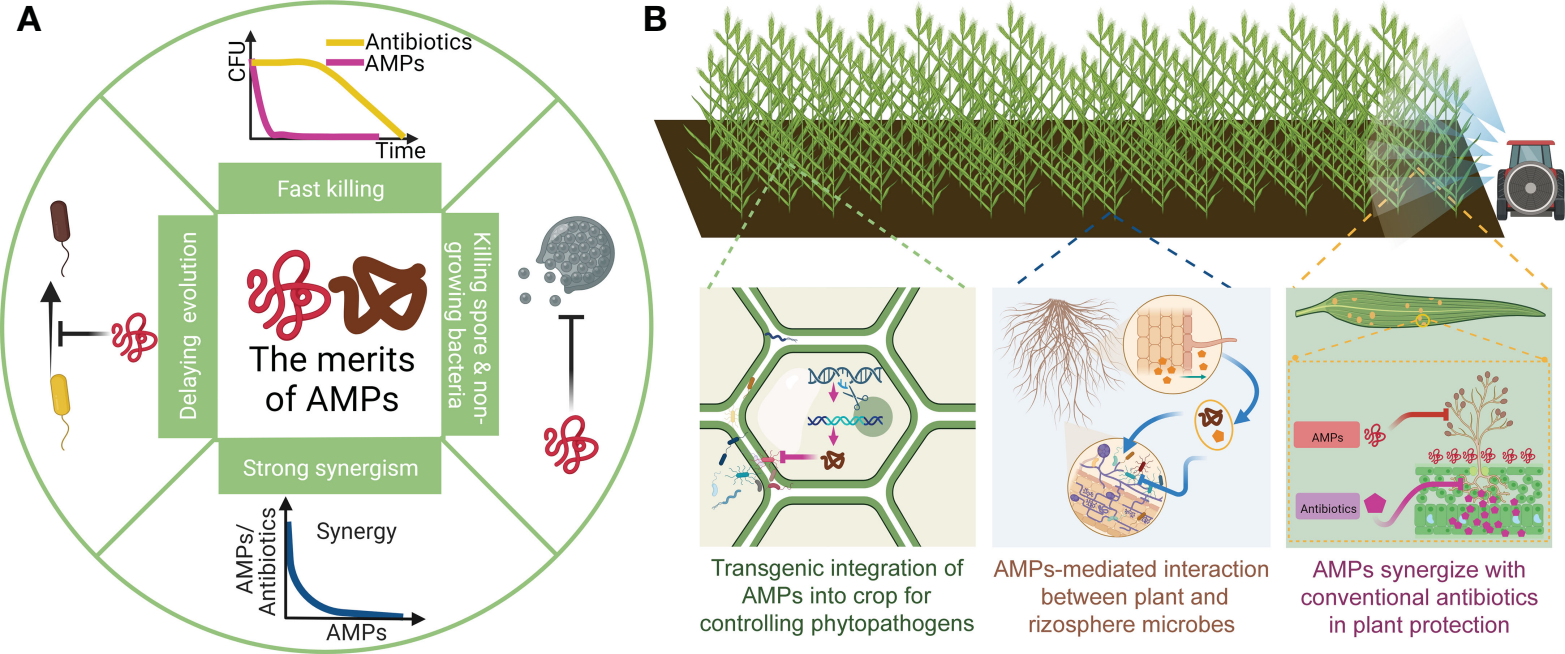
The merits of AMPs and their application in plant protection. **(A)** An brief illustration of AMPs’ merits. AMPs are able to rapidly kill bacteria within minutes. Due to distinct killing mechanism, they can deactivate non-growing bacterial and fungal spores. In addition, they also broadly synergize with other AMPs and antibiotics. Last, resistance evolution of pathogens to AMPs are particularly slow with high fitness cost. **(B)** AMPs with above merits can play an important role in integrated pathogen management. An integrated management methods includes endogenously expressing AMPs in plant to directly kill invading pathogens and to regulate symbiosis and root microbiome. Also, spraying AMPs in crop field with conventional antibiotic can form a synergistic effect that more efficiently eliminates pathogens, reduces the use of conventional antibiotics as well as delays pathogen resistance.

AMPs have long been proposed as a potential anti-bacteria and anti-fungi reagents in agriculture (Van der Biezen, 2001; Montesinos, 2007; Marcos et al., 2008; Montesinos and Bardají, 2008; Wang et al., 2018; Lobo and Boto, 2022), medicine (Zasloff, 2002; Harris and Coote, 2010; Tam et al., 2015; Hayouka et al., 2017; Annunziato and Costantino, 2020), food industry (Gupta and Srivastava, 2014; Sibel Akalın, 2014; McNulty et al., 2019; Lima et al., 2021; Liu et al., 2021). Here we briefly summarize the general information of AMPs and provide references for readers that are not familiar with AMPs. Most of the AMPs are usually composed of 10 to 50 amino acids with a total net positive charge in the working environments. These amino acids form different secondary structures, namely α-helix, β-sheet, macrocycles and residual-modified structures, which are the important bases of AMPs’ classification (Zasloff, 2002). Also, classification of AMPs in plants is characterized by the disulfide bonds and tertiary structures (Tam et al., 2015). In addition, other standards, such as pathogens that AMPs target, also can be applied to categorize AMPs, i.e. antifungal AMPs and antiviral AMPs (Benfield and Henriques, 2020). AMPs from different origins share similar killing mechanisms when targeting bacterial and fungal pathogens. As summarized by many previous reviews (Zasloff, 2002; Brogden, 2005; Melo et al., 2009; Bocchinfuso et al., 2011; Sibel Akalın, 2014; Benfield and Henriques, 2020; Huan et al., 2020), most of AMPs directly target and rupture the cell membrane to kill pathogens, only a few AMPs enter the cytoplasm to interrupt the cellular physiological process (Rahnamaeian et al., 2015). In general, the membrane-targeting mechanisms of AMPs can be characterized by carpet models, toroidal pore models and barrel-stave models (Epand and Vogel, 1999; Zasloff, 2002; Benfield and Henriques, 2020). Although many previous studies mostly focused on the design, classification, physical structures and chemical properties, as well as killing mechanism of AMPs, which are generally shared by many different AMPs for various usages. We, however, propose that more information and properties of AMPs should be considered especially when using AMPs for plant protection. In this minireview, we attempt to discuss some of the overlooked properties of AMPs including steep dose-response relations, fast killing, slow resistance selection, broad synergism. It should be noted that many of these properties are not discovered in the field of plant protection. For example, the fast killing property of AMPs was revealed by researchers who study biological nano-materials (Fantner et al., 2010), and the slow resistance selection property was discovered by research groups that study ecology and evolution (Perron et al., 2006; Yu et al., 2018; Lazzaro et al., 2020). These properties directly relate to the dosing strategy and effectiveness in different scenario of application (Jensen et al., 2022). We therefor also highlight how these distinctly overlooked properties can be exploited and integrated into the pathogen management protocols (See **Figure 1**).

## AMPs protect plant from infections both inside and outside

The immune repository of higher plant contains a group of antimicrobial peptides. Most of the plant AMPs are cystine-rich peptides and cross-linked by disulfide bonds (Tam et al., 2015; Sathoff and Samac, 2019). These plant AMPs, in general, are not structurally different from those from animals. Based on differences in disulfide bonds and tertiary structures, these peptides can be classified into several families, which includes thionins, defensins, hevein-like peptides, knottin-like peptides, lipid transfer proteins and snakins (Tam et al., 2015). Plant antimicrobial peptides widely expressed in variety of plant organs at different developmental stages (Silverstein et al., 2007; Tesfaye et al., 2013), which can be up-regulated upon pathogen infection. AMPs isolated from immune-activated plant showed robust killing effect towards various fungi and bacteria (Segura et al., 1999; Kovalskaya and Hammond, 2009). Overexpression of several defensin-like peptides in plants confer stronger antifungal and antibacterial effect. Moreover, plant defensin peptides both in model and crop plants have shown enhanced and long lasting disease resistance.

Heterologous expression of AMPs in plant using transgenic technologies is one of the efficient and powerful practices to improve plant’s resistance to phytopathogens. The full-length of cDNA of target AMPs was fused to the carrier plasmid, such as pMON22659 and pSAI4 and then transformed in to target plant using Agrobacterium. With this method, researcher integrated alfalfa antifungal peptide (alfAFP) defensin into potato and achieved robust resistance to fungal pathogens *Phytophthora cactorum* and *Fusarium solani*, the bacterium *Erwinia carotovora* both in greenhouse and field (Gao et al., 2000), where the transgenic plant had sixfold lower fungal load. Expression of a variety of plant and animal peptides in plant also confers both bacterial and fungal resistance in different families of plant, including potato (Gao et al., 2000; Osusky et al., 2000), tomato (Chan et al., 2005), rice (Kanzaki et al., 2002), tobacco (Chakrabarti et al., 2003; Swathi Anuradha et al., 2008; Khademi et al., 2020; Zhou et al., 2021), banana (Chakrabarti et al., 2003; Ghag et al., 2012), cotton (Gaspar et al., 2014), and many more other plants in earlier reviews (Keymanesh et al., 2009; Iqbal et al., 2019). This method can potentially reduce the infection and transmission of plant pathogens, and also decrease the incidence of pathogen infection of co-planted non-transgenic plants. Expression of heterologous AMPs in plants requires extra energy and resources and often trade-off with other traits of the plant, such as the yield vegetative growth (Van der Biezen, 2001; Jin et al., 2005). Therefore, it is still awaiting to evaluate that how the environmental factors, such as drought, temperature and many other biotic factors, affect the overall and long-term effect regarding disease resistance in transgenic plants. In addition, evolutionary theory predicts that enhanced host immunity selects more virulent pathogens (Antia et al., 1994; Cressler et al., 2014). Pathogens can evolve, if not direct resistance to host immune effectors, higher virulence and pathogenicity to reduce the expression of immune functions, and eventually break down the disease resistance (Sacristán and García Arenal, 2008; Fleming-Davies et al., 2018). Such breakdown of disease resistance has occurred in the cultivars that integrated R genes (Dodds et al., 2006; Peressotti et al., 2010). It remains elusive that if the transgenic plant with enhanced AMPs expression select virulent pathogens strains that compromises the durable resistance.

*In-vitro* application of AMPs can be also an effective way to control plant disease. Due to the costly production of AMPs at the moment, large scale field studies and applications of AMPs are rare (**Table 1**). The synthetic antimicrobial peptide BP15 showed great potential in controlling brown spot disease of pear, showing a disease reduction of about 42%-60% in the serial trials (Puig et al., 2015). Hayouka et al., 2017 synthesized 20-amino-acid-length peptides chain mixture with only two kinds of amino acids with relatively low cost. Those random peptides mixtures are able to rapidly eradicate pathogenic bacteria in plants (Topman et al., 2018; Amso and Hayouka, 2019). Foliar spray of these peptides significantly reduces disease incidence and index, which is almost as effective as commercial copper-based bactericide. In addition, these AMP mixtures showed no toxic effect on beneficial insects and mammalian cells. These studies show that synthesized AMPs have great potential in plant protection. Another possible way is to apply those widely used AMPs to control plant pathogens. For example, the widely-used food preservative, ϵ-poly-L-lysine (ϵ-PL), can successfully control grey mould on tomato in the field (Sun et al., 2017; Sun et al., 2018). Application of peptaibol trichogin with very low concentration (100 µM/129.3 g/ha) in vineyard has significantly reduced the incidence and severity of grapevine downy mildew caused by *Plasmopara viticola* (Bolzonello et al., 2023). Moreover, the peptide has no phytotoxicity on the plant.

**Table 1:** The peptides used for controlling plant pathogens and their scale of testing.

Peptides	Origin	Targeting pathogens	Methods of testing	Scale of testing	Refs
Cecropin A	*Hyalophora cecropia*	*Fusarium oxysporum*, *Dickeya dadantii*, *Fusarium verticillioides*	*In-vitro* killing assay, Transgenic expression,	lab scale, Green house scale	(Cavallarin et al., 1998; Bundó et al., 2014; Montesinos et al., 2016)
Snakin-1	Potato	*Clavibacter michiganensis*, *Botrytis cinerea*	Transgenic expression	lab scale	(Segura et al., 1999)
MsrA1	Synthetic	*Phytophthora cactorum*, *Fusarium solani*, *Erwinia carotovora*, *Alternaria brassicae*, *Sclerotinia sclerotiorum*	Transgenic expression	Lab scale, Greenhouse scale	(Osusky et al., 2000; Rustagi et al., 2014)
alfAFP	alfalfa	*Verticillium dahliae*,	Transgenic expression	Lab scale, Greenhouse scale, Field scale	(Gao et al., 2000)
MSI-99	Synthetic	*Fusarium oxysporum*, *Sclerotinia sclerotiorum, Alternaria alternata*, *Botrytis cinerea*	Transgenic expression	Lab scale, Greenhouse scale	(Chakrabarti et al., 2003)
Thionin	*Arabidopsis thaliana*	*Ralstonia solanacearum*, *Fusarium oxysporum*	Transgenic expression	Lab scale	(Chan et al., 2005)
BP/BPC serial peptides	Synthetic	*Erwinia amylovora, Xanthomonas vesicatoria*, *Pseudomonas syringae*, *Fusarium oxysporum, Penicillium expansum, Aspergillus niger*, *Rhizopus stolonifer*, *Stemphylium vesicarium*	*In-vitro* killing assay, Foliar spray, Transgenic expression	Lab scale, Greenhouse scale, Field scale	(Monroc et al., 2006; Badosa et al., 2007; Badosa et al., 2009; Güell et al., 2011; Nadal et al., 2012; Puig et al., 2014; Puig et al., 2015; Montesinos et al., 2017; Mariz-Ponte et al., 2021; Montesinos et al., 2021)
NmDef02	*Nicotiana megalosiphon*	*Peronospora hyoscyami*	*In-vitro* killing assay, Transgenic expression	Lab scale, Greenhouse scale, Field scale	(Portieles et al., 2010)
hCAP18/LL-37	Human	*Pectobacterium carotovorum*	*In-vitro* killing assay, Transgenic expression	Lab scale	(Jung et al., 2012; Holásková et al., 2018)
Thanatin	*Podisus maculiventris*	*Fusarium graminearum*, *Botrytis cinerea*	*In-vitro* killing assay, Transgenic expression	Lab scale	(Koch et al., 2012)
Tachyplesin I	Horseshoe crab	*Pectobacterium carotovorum*	*In-vitro* killing assay, Transgenic expression	Lab scale	(Lipsky et al., 2016)
Melittin	*Apis mellifera*	*Xanthomonas oryzae*	*In-vitro* killing assay	Lab scale	(Shi et al., 2016)
Tannins	*Sapium baccatum*	*Ralstonia solanacearum*,	*In-vitro* killing assay, Foliar spray	Lab scale, Greenhouse scale	(Vu et al., 2017)
ϵ-poly-L-lysine	Synthetic	*Botrytis cinerea*	*In-vitro* killing assay, Foliar spray	Lab scale, Greenhouse scale, Field scale	(Sun et al., 2017; Shu et al., 2021)
Random peptides mixture	Synthetic	*Xanthomonas perforans*, *Xanthomonas campestris*,	*In-vitro* killing assay, Foliar spray	Lab scale, Greenhouse scale	(Topman et al., 2018)
PAF26	Synthetic	*Penicillium digitatum*	*In-vitro* killing assay	Lab scale	(Wang et al., 2018)
NoPv1	Synthetic	*Plasmopara viticola*, *Phytophthora infestans*	*In-vitro* killing assay, Foliar spray	Lab scale, Greenhouse scale	(Colombo et al., 2020)
Ac-AMP2	*Amaranthus caudatus*	*Penicillium expansum*	*In-vitro* killing assay, Transgenic expression	Lab scale	(Huang Y et al., 2021)
SAMP	*Microcitrus australiasica*	*Liberibacter crescens*	*In-vitro* killing assay, Transgenic expression, Foliar spray	Lab scale, Greenhouse scale	(Huang C et al., 2021)

## AMPs rapidly kill pathogens in concentrations above the threshold

The fast-killing property of AMPs is vital for its application and often overlooked. Boman firstly recorded that the fast elimination of bacterial pathogens in immune-activated *Drosophila* (Boman et al., 1972). They found that the “vaccinated” flies with frozen bacteria can kill more than 99.9% of bacteria cells within 7 min after the second infection, then reduce the bacterial load for several orders of magnitudes in next few minutes. Boman and colleagues later proved that the fast killing is mainly achieved by AMPs that expressed by the insect’s immune system (Hultmark et al., 1980). Similar fast-killing property was constantly observed in later isolated AMPs, such as Magainins, the peptide isolated from frog skin (Zasloff, 1987). High-speed atomic force microscope allows one to observe the killing process of AMPs in real time (Fantner et al., 2010). In sufficient high concentrations, AMP can kill bacteria within 30 seconds. Similar fast killing rate was also observed using time-lapse fluorescence microscopy (Barns and Weisshaar, 2013). In contrast, antibiotics take much longer to kill bacteria. For example, bactericidal beta-lactam antibiotics take nearly one hour to kill a single bacterium (Yao et al., 2012). The killing time of many other antibiotics in bulk bacterial culture can range from 12 hours to several days (Ferro et al., 2015; Yang et al., 2018; Vaddady et al., 2019). It’s thus evident that the killing rate of AMPs is order-of-magnitude faster than that of antibiotics.

In addition, AMPs show striking inoculum effect (Nannini et al., 2013; Lenhard and Bulman, 2019; Loffredo et al., 2021). In other words, the killing effects of AMPs depends both on the bacterial density and AMP concentration, i.e. a minimal number of AMPs molecules is required to kill a bacterium (Huang, 2006; Melo et al., 2009). According to quantitative estimation, it requires approximate 10^7^ peptides per cell for PMAP-23 to kill bacteria in bulk culture (Roversi et al., 2014). Moreover, AMP molecules can be unevenly absorbed by bacterial population, which results delayed population growth (Snoussi et al., 2018). Dead bacteria can even bind AMPs molecules and cause the same effect (Wu and Tan, 2019). This indicates that fast depletion of AMPs molecules by some bacterial cells results decreased free AMPs molecules in solution, and protect other bacteria from killing.

The fast-killing and inoculum effect altogether shapes the steep dose-response curve (Yu et al., 2016). It is notable that the concentration range from no effect to full killing is roughly 10 times in various AMPs (Steiner et al., 1981; Zasloff, 1987; Thevissen et al., 1999; Yu et al., 2016; Savini et al., 2017; Savini et al., 2020). This implies that we need to quantitatively evaluate the effective concentration when AMPs are applied in agricultural practice (Mercer et al., 2020; Jensen et al., 2022). Very recently, researchers have borrowed the methods in pharmacokinetics to quantify the spatial and temporal dynamics of antibiotic concentrations used to treat Huanglongbing in citrus (Li et al., 2019; Li et al., 2021). Moreover, the benchmark dose modelling method has also been proposed to quantify the working concentration of pesticides in the field (Jensen et al., 2022).These studies helped design proper dosing strategies in plant protection, which is also applicable for using AMPs in plant protection.

## AMPs slowly select mutants with large fitness cost

Bacteria evolve resistance to all antimicrobial agents, but the evolution in AMPs is particularly slow. Experimental evolution revealed that the resistance evolution in AMPs is averagely 10 times slower than in antibiotics (Dobson et al., 2013; Spohn et al., 2019). This can be explained by the AMP’s steep threshold-like dose-response curve (Melo et al., 2009; Yu et al., 2018). When the concentration is higher than the “threshold”, the exposed bacterial can be immediately eliminated (Fantner et al., 2010). If the concentration is lower than the “threshold”, AMPs neither impose physiological stress on bacteria cells nor arrest their growth (Rodríguez-Rojas et al., 2014; Snoussi et al., 2018). It implies that the concentration range that select resistant mutants is rather narrow (Yu et al., 2018).

Moreover, resistance to AMPs often suffers significant fitness cost in bacteria. Resistance to AMPs in bacteria is generally caused by the loss of lipids or modification of lipid A on the membrane (Pränting et al., 2008; Andersson et al., 2016; Gao et al., 2016), which substantially impedes nutrition uptake and attenuate bacterial growth. For example, *Salmonella typhimurium* strains that resistant human peptide LL-37 suffers 25% of growth reduction compare to the wildtype control (Lofton et al., 2013). Colistin-resistant *Acinetobacter baumannii* strains have fitness cost ranging from 10% to as high as 60% (Mu et al., 2016). High fitness cost makes resistant strains less competitive in low or no antibiotic condition. Bacteria harboring *mcr-1* gene can be completely removed within 20 days during thermophilic composting in environment (Gao et al., 2019). Large scale epidemiological survey showed that the frequency of bacteria carrying colistin-resistant genes immediately decreased after banning of colistin in feeding industry as well as in clinics (Shen et al., 2020; Wang et al., 2020; Shen et al., 2021).

## AMPs synergize with other antimicrobials and plant immune system

Higher plants deploy a combination of antimicrobial peptides to combat the pathogen invasion. The cocktail of AMPs can be synergistic at different organizational level. AMPs not only synergize with themselves, but also interact with plant host physiological process to eliminate pathogen infection. Here, we categorize the synergism of AMPs into molecular synergism and functional synergism.

Molecular synergism shows that AMPs directly cooperating with other antimicrobials in killing pathogens. Plant AMPs 2S albumins synergize with thionins in inhibiting fungal growth and achieved 2- to 73-fold of increased killing effect compared to single AMP (Terras et al., 1993). Similar synergistic effects among AMPs from different organisms were further observed in eliminating bacteria, fungi and parasites (Westerhoff et al., 1995; McCafferty et al., 1999; Yan and Hancock, 2001; Marxer et al., 2016; Yu et al., 2016). In addition, AMPs also synergize with other antibiotics and fungicides (Arikan et al., 2002; Gonzalez et al., 2002; Mariz-Ponte et al., 2021). Such synergistic combination effect among AMPs can substantially reduce the total amount of molecules that required to kill the pathogen. This implies that application of AMPs in plant protection can reduce the use of agricultural antibiotics and fungicides, as well as the total cost of crop production. Moreover, the combination effect of AMPs can also delay drug resistance evolution and prolong the duration of agricultural chemicals (Maron et al., 2022).

Moreover, synergistic effect of killing can be also achieved by targeting different development stages of pathogens. Most of the agricultural antibiotics and fungicides only act on pathogens on the growing stage, not on the non-growing stage, i.e. the dominant spores. Previous researches showed that AMPs killed non-germinating fungal spores (Levitz et al., 1986; Rioux et al., 2000; Velivelli et al., 2020), thus can substantially reduce the transmission of pathogens. Moreover, spore elimination is particularly important in greenhouse agriculture. Air spora is the main source of plant disease in greenhouse. High density of spore also threatens the health of workers in greenhouse (Ercilla-Montserrat et al., 2017; Madsen et al., 2021). AMPs can be used as a sporicidal to inhibit transmission of plant disease and to protect the health of greenhouse workers. Thus, AMPs can potentially synergize with other antimicrobials, by eliminating the air spora, to reduce the disease prevalence and health risks of agricultural workers.

Functional synergism of AMPs in targeting pathogen is the result of complex interaction between AMPs and pathogen’s physiology. The positively charged AMPs with membrane permeability are able to not only attach on pathogens’ membrane but also target intracellular components of pathogens, which drastically accelerate the killing process. An insect antimicrobial peptide, abaecin, can interact with chaperones in bacteria, which can substantially dampen the bacterial stress response. It enhanced the killing effect of hymenoptaecin for four-fold in terms of inhibition rate, a strong synergistic effect in that combination (Rahnamaeian et al., 2015). In addition, *in-vitro* assay shows that plant AMPs modulate cellular redox stress in fungi by inducing the accumulation of ROS in vegetative cells and ultimately trigger apoptosis (Van der Weerden et al., 2008; Mello et al., 2011). Moreover, AMPs also activate the MAPK signaling cascade in fungal pathogens to control its growth (Ramamoorthy et al., 2007). Such synergism in fighting against pathogens can also be achieved by AMPs in interacting with plant signal pathways. For example, the cysteine-rich protein designated defensin, SPD1, in sweet potato both regulates the redox status of ascorbate and inhibit fungal and bacterial pathogens (Huang et al., 2008). Plant AMPs also interact with many other regulating pathways to enhance the plant defense and achieve synergism in controlling pathogens (Bolouri Moghaddam et al., 2016).

AMPs directly interacting with plant immune system can be defined as another functional synergism. However, experimental evidence is rare. Previous studies showed that endogenously expressed AMPs can bind with some transcription factors and regulate many signaling pathways (Damon et al., 2012). The consequence of these interactions is not revealed yet. Exogenous expression of a designed AMPs in plant will not elicit the immune response (Badosa et al., 2017; Montesinos et al., 2021), and the plant immunity can only be elicited when that AMPs conjugated with plant immune eliciting peptides, such as flg-15 and flg-22 (Montesinos et al., 2021). In a recent study, a stable antimicrobial peptides from *Microcitrus australiasica*, the Australian finger lime, was developed to treat Huanglongbing in citrus (Huang C et al., 2021). This peptide, also named as MaSAMP, not only directly target and kill the main pathogens *Liberibacter crescens* of citrus tree, but also induce plant’s innate immunity to prevent and inhibit infections. Both foliar spray and pneumatic truck injection of MaSAMP to HLB-infected citrus trees significantly reduced disease severity and bacterial load. Moreover, the authors found that MaSAMP application can induce the expression of a set of defense genes, such as pathogenesis-related proteins and the enzyme of SA biosynthesis and phenyl propanoid pathways. This peptide can broadly activate systemic defense responses in tobacco, tomato and citrus trees. This study demonstrated that AMPs can serve bifunctionally as both pathogen eliminators and stimulants of plant immune responses. Such synergistic functions can better protect plant from infections and shows great potential in plant protection.

## AMPs regulate symbionts of plant

The nodule-specific cysteine-rich (NCR) AMPs also play an important role in mediating plant-microbe interaction inside the legume plants. These antimicrobial peptides can both maintain and eliminate the viability of nitrogen-fixing bacteroids. At the initiation stage of root nodule, lytic AMPs kill most of the phytopathogenic fungi and bacteria, and maintain the symbiotic bacteria inside root. NCR211 expressed in the nodule interzone II-III promotes differentiation of bacteroids and the formation of nodules. Synthetic NCR211 was further proven inhibiting aggregation of free-living *S. meliloti* (Kim et al., 2015). In legume plants, nitrogen-fixing symbiotic bacterioids with medium population size can significantly promote the growth of host plant by fixing nitrogen for the host plants. It, however, can also retard host’s growth when the load of bacteroids is high (Sachs et al., 2018). Inside the roots of host plants, a variety of nodule-specific cysteine-rich peptides are secreted to regulate the population of nitrogen-fixing rhizobia (Van de Velde et al., 2010; Wang et al., 2010). Knockout of host’s nodule-specific cysteine-rich peptide demonstrated that these peptides may serve as the extractors of the host plant to harvest nitrogen nutrients synthesized by the bacteroids (Wang et al., 2017; Yang et al., 2017). The direct killing effect of nodule-specific cysteine-rich peptides was measured *in vitro* as well (Van de Velde et al., 2010). Moreover, these peptides can enter bacterial cytosol and bind with intracellular molecules to slow down the growth of these bacteroids. The balance between plant host and symbionts may be the result of the complex interaction between AMPs and other host peptides (Yang et al., 2017).

All together, these evidence indicates that host plant may harness host peptides to finely tune the symbiosis at different temporal and spatial scale. It is also intriguing that whether these antimicrobial host peptides can be genetically incorporated into non-legume plants to develop symbiotic associations (Pankievicz et al., 2019). In addition, it remains elusive that if these peptides can be secreted by roots and released into soil for mediating broader host-microbe interaction.

## Challenges and perspective

Crop yield loss is mainly caused by plant pathogen and pests. The total loss can reaches up to 40% of crop yield at the global level according to a recent survey (Savary et al., 2019), which is apparently far beyond the compensation of any advanced breeding technologies that contributed the yield gain around only 1-3% per year (Tester and Langridge, 2010). This indicates that the most efficient way to promote crop yield is to prevent it from the loss caused by pests and pathogens. Due to the diversity of pathogens at various levels, integrated management strategies are urgently needed to protect the crop. In this short review, we propose that AMPs with key merits can be harnessed as an important part in the integrated strategy.

In design of plant protection strategy, no single component or method can accomplish all of the tasks. We argue that an optimal and cost-effective method that makes full use of AMPs’ merits can form a better solution for plant pathogen management (**Table 2**). With the fast-killing property, AMPs nearly take no time to kill the pathogens. This makes AMPs quickly fulfilling their functions before inactivated by environmental factors. Although the large molecular weight of AMPs may weaken their systemic effects for eliminating infection inside plants, One can combine AMPs with conventional antimicrobials in order to achieve systematic synergistic effect in control plant pathogens. In this case, AMPs eradicate the planktonic bacterial pathogens and fungal spores on the surface of plants in order to stop the spreed of plant disease in the field. The conventional antimicrobials with stronger systemic effects kill pathogen that grows inside plants. In addition, heterologous express of AMPs using transgenic plants is also an option for fighting against phytopathogens. Moreover, the sharp dose-response relation of AMPs suggests that field application with low concentrations may completely fail to kill plant pathogen. It is important that the concentration of AMPs sprayed on plant should be higher than the working critical threshold.

**Table 2:** The pros and cons of using antimicrobial peptides to treat plant pathogens.

Advantages	Disadvantages
AMPs rapidly kill both fungal and bacterial pathogens of all stages including spores.	AMPs have weakly systemic effect cannot eliminate pathogens inside host plant.
AMPs slowly select resistant pathogens.	AMPs have strong inoculum effect, which means the working concentration need to be carefully determined.
AMPs can be easily integrated into plant using transgenic transformation.	Expression of AMPs in plant consumes the resources used for growth and reduce the yield.
AMPs synergize with many other antimicrobials.	Production of AMPs is still relatively expensive.
AMPs manipulate the symbionts inside plant and promote plant growth.	Long-terms of exposure of AMPs may select resistant pathogens.

Antibiotic resistance of plant pathogen is always a concern for agricultural production as well as human health. The direct evidence of transfer of resistance pathogen from farm to clinics is rare (Chang et al., 2015). Both experiments and theories proved that AMPs slowly select resistant pathogens with high fitness cost. This indicates that resistant strains can be quickly out-competed by its sensitive counterpart. Seasonal application of AMPs should be less of a concern for the resistance evolution. We also argue that combined application of AMPs with conventional antibiotics not only adds up the overall effect controlling, but also slow down pathogen’s resistance evolution towards conventional antibiotics (Lazzaro et al., 2020)

Large scale application of AMPs requires highly cost-effective production. Recently technological advancement allows one to cheaply produce AMPs either through solid-phase synthesis (Hayouka et al., 2017; Topman et al., 2018), prokaryotic expression (Zhao et al., 2015; Schreiber et al., 2017) and eukaryotic expression (Holaskova et al., 2015; Holásková et al., 2018). Besides, some AMPs, such as Polylysine, that used in food and livestock industry are also ready for plant protection. Although many lab and greenhouse experiments have proven that AMPs are effective against various phytopathogens, one still anticipates large scale field tests in various environmental conditions.

## Author contributions

All authors listed have made a substantial, direct, and intellectual contribution to the work, and approved it for publication.
